# Integrative Transcriptomic Analysis Uncovers Novel Gene Modules That Underlie the Sulfate Response in *Arabidopsis thaliana*

**DOI:** 10.3389/fpls.2018.00470

**Published:** 2018-04-10

**Authors:** Carlos Henríquez-Valencia, Anita Arenas-M, Joaquín Medina, Javier Canales

**Affiliations:** ^1^Instituto de Bioquímica y Microbiología, Facultad de Ciencias, Universidad Austral de Chile, Valdivia, Chile; ^2^Instituto de Producción y Sanidad Vegetal, Facultad de Ciencias Agrarias, Universidad Austral de Chile, Valdivia, Chile; ^3^Centro de Biotecnología y Genómica de Plantas, Instituto Nacional de Investigación y Tecnología Agraria y Alimentaria, Universidad Politécnica de Madrid, Madrid, Spain; ^4^Millennium Institute for Integrative Systems and Synthetic Biology (MIISSB), Santiago, Chile

**Keywords:** gene co-expression analysis, sulfur, sulfate assimilation, microarray analysis, network analysis, transcription factors

## Abstract

Sulfur is an essential nutrient for plant growth and development. Sulfur is a constituent of proteins, the plasma membrane and cell walls, among other important cellular components. To obtain new insights into the gene regulatory networks underlying the sulfate response, we performed an integrative meta-analysis of transcriptomic data from five different sulfate experiments available in public databases. This bioinformatic approach allowed us to identify a robust set of genes whose expression depends only on sulfate availability, indicating that those genes play an important role in the sulfate response. In relation to sulfate metabolism, the biological function of approximately 45% of these genes is currently unknown. Moreover, we found several consistent Gene Ontology terms related to biological processes that have not been extensively studied in the context of the sulfate response; these processes include cell wall organization, carbohydrate metabolism, nitrogen compound transport, and the regulation of proteolysis. Gene co-expression network analyses revealed relationships between the sulfate-responsive genes that were distributed among seven function-specific co-expression modules. The most connected genes in the sulfate co-expression network belong to a module related to the carbon response, suggesting that this biological function plays an important role in the control of the sulfate response. Temporal analyses of the network suggest that sulfate starvation generates a biphasic response, which involves that major changes in gene expression occur during both the early and late responses. Network analyses predicted that the sulfate response is regulated by a limited number of transcription factors, including MYBs, bZIPs, and NF-YAs. In conclusion, our analysis identified new candidate genes and provided new hypotheses to advance our understanding of the transcriptional regulation of sulfate metabolism in plants.

## Introduction

Sulfur (S) is an essential nutrient for living organisms, including microorganisms, animals, and plants. Animals are unable to assimilate inorganic S sources and must obtain this nutrient from organic compounds such as proteins in their diet. This source of organic S ultimately depends on the ability of plants to assimilate S from inorganic sources, highlighting the importance of plants in the natural global S cycle (Takahashi et al., 2011). Plants have a constitutive demand for S driven by the need to synthesize essential S-containing compounds, such as amino acids [cysteine (Cys) and methionine], glutathione, and secondary metabolites, all of which help to sustain biological processes related to growth, development and defense (Maruyama-Nakashita, 2017).

The main source of S for plants is the sulfate anion (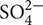), which is taken up by root epidermal cells via the activity of specific sulfate transporters (SULTRs), mostly SULTR1;1 and SULTR1;2 (Yoshimoto et al., 2002). From the root tissue, sulfate is then transported to the aerial portion of plants through SULTRs such as SULTR2;1 and SULTR3;5 (Takahashi et al., 2000; Kataoka et al., 2004a). In addition to its long-distance transport, sulfate can be stored in the vacuoles and the release from these organelles is facilitated by the activities of the sulfate transporters SULTR4;1 and SULTR4;2 (Buchner et al., 2004; Kataoka et al., 2004b). Interestingly, the expression of genes coding for these sulfate transporters is induced by S starvation (Maruyama-Nakashita, 2017). The first step in sulfate assimilation occurs in the chloroplasts or cytosol, where a reaction catalyzed by isoforms of the enzyme ATP sulfurylase (ATPS) generates the metabolite adenosine 5′-phosphosulfate (APS). APS is one of the key intermediaries of the sulfate assimilation pathway and represents a branching point of that pathway (Kopriva et al., 2012). APS can be phosphorylated by the activity of APS kinase (APK), generating 3′-phosphoadenosine-5′-phosphosulfate (PAPS), an essential metabolite for the biosynthesis of sulfated compounds such as glucosinolates (GSLs) (Koprivova and Kopriva, 2016). GSLs have an important function in the defense of plants against pests and pathogens, especially in the Brassicales (Halkier and Gershenzon, 2006). APS can also be reduced to sulfite by the enzyme APS reductase (APR) in the chloroplasts; in turn, sulfite is reduced to sulfide by sulfite reductase (SiR) (Gotor et al., 2015). Sulfide is then incorporated into *O*-acetylserine (OAS) to form Cys (Gotor et al., 2015). Cys is a branching point in this pathway, leading to methionine biosynthesis or glutathione biosynthesis (Romero et al., 2014). Alternatively, by using sulfide as a source of S, mitochondria can also synthesize Cys (Romero et al., 2014). Cys biosynthesis requires the additional coordination of nitrogen and carbon metabolism, representing a key point for the dynamic control of these important metabolic pathways (Hesse et al., 2004). Recent evidence indicates that the availability of Cys precursors is perceived by plant cells via the GNC2 and TOR signaling pathways (Dong et al., 2017). In summary, sulfate assimilation involves the coordination of multiple subcellular organelles and enzymes, suggesting that complex regulatory mechanisms are involved in the regulation of the S metabolic pathway.

During the past few decades, as a consequence of the reduction in industrial S emissions, S deposition in the soil has severely decreased (McNeill et al., 2005). Lower S availability can reduce crop yields and impact the quality of harvested products (McGrath and Zhao, 1996; Bonnot et al., 2017). S deficiency also can impact the internal levels of important nutrients such as nitrate, phosphate, molybdenum, and iron (Forieri et al., 2017). Therefore, S starvation is problematic in modern agriculture in several industrialized countries.

Several studies have reported that S limitation triggers regulatory metabolic systems, such as the catabolism of S-storage compounds and suppression of the biosynthesis of secondary S metabolites (Maruyama-Nakashita, 2017). By entirely rearranging the flow of S metabolism, plants sustain their growth in low-S environments (Maruyama-Nakashita, 2017). This rearrangement involves the activation of sulfate uptake by inducing the expression of high-affinity sulfate transporters, the modification of GSL metabolism and the dramatic accumulation of OAS, the last of which is a signaling compound for responses to the lack of S (Nikiforova et al., 2003; Hubberten et al., 2012).

The complex signaling network of sulfate assimilation in plants is regulated by several transcriptional and post-transcriptional mechanisms (Maruyama-Nakashita, 2017). At the transcriptional level, S starvation induces the expression of several SULTRs via specific S-responsive *cis*-acting elements (SUREs) in their promoter regions (Maruyama-Nakashita et al., 2005; Rouached et al., 2008). Moreover, a post-transcriptional regulatory mechanism mediated by the activity of microRNA-395 (miR-395), which regulates several genes of the sulfate assimilation pathway in response to S limitation, has also been reported (Kawashima et al., 2009). In the past few decades, multiple efforts have been carried out to identify factors regulating the S-starvation response: SLIM1 (sulfur limitation1) (Maruyama-Nakashita et al., 2006), HY5 (Lee et al., 2011), miR-395 (Kawashima et al., 2009), several MYB transcription factors (TFs) (Celenza et al., 2005; Gigolashvili et al., 2007, 2008; Hirai et al., 2007), and SDI1 (sulfur deficiency induced 1) (Aarabi et al., 2016) as well as other factors related to epigenetic mechanisms such as SHM7 (Huang et al., 2016). Despite these recent advances, the molecular mechanisms that control S-starvation responses have only slowly began to emerge (Koprivova and Kopriva, 2014).

In the past decade, a considerable amount of transcriptomic data has been generated for the model plant *Arabidopsis thaliana*; these data correspond to a wide range of different tissues, developmental stages, stress conditions, and genotypes (Zimmermann et al., 2008). Analysis of this valuable information can provide information that cannot be gained by analyzing individual experiments (Rest et al., 2016). In the past few years, several transcriptomic analyses of the response of *A. thaliana* plants to sulfate starvation have been reported (Maruyama-Nakashita et al., 2005; Iyer-Pascuzzi et al., 2011; Bielecka et al., 2015; Aarabi et al., 2016; Forieri et al., 2017). By using an integrative meta-analysis of transcriptomic data from sulfate-availability experiments of different laboratories, we aimed to explore the unknown gene expression networks underlying the sulfate response in *A. thaliana* in this paper. Our results suggest that transporters and enzymes related to sulfate assimilation are consistently transcriptionally regulated by sulfate availability and reveal biological processes that have not been extensively studied in the context of the sulfate response, including cell wall organization, the regulation of proteolysis and carbon/nitrogen metabolism. In addition, we identified new regulatory factors, such as bZIP1, RVE2 and NF-YA2, that might be involved in the control of the S-starvation response. Combining a meta-analysis and functional analyses of candidate genes together will contribute to an improved understanding of the biology of sulfate responses in plants.

## Materials and Methods

### Plant Materials and Growth Conditions

*Arabidopsis thaliana* ecotype Columbia (Col-0) was used in this study. Seeds were sterilized with 50% bleach (v/v) and 0.1% Triton X-100, after which they were rinsed with sterilized water at least four times. The seeds were stratified at 4°C for 24 h in darkness and then germinated on plates containing Murashige and Skoog (MS) medium that was supplemented with 1% sucrose and 0.6% agar. The plates were incubated in a growth chamber (Bioref-19 incubator, PiTec, Santiago, Chile) at 23°C under a 16/8-h photoperiod and a light intensity of 125 μmol m^-2^ s^-1^ for 5 days. At dawn on the fifth day, the seedlings were transplanted to 6-well plates that contained liquid MS medium [full nutrient (FN)] or modified liquid MS medium that lacked sulfate, after which they were allowed to grow for 2 days. Afterward, the 7-day-old seedlings were treated with either 1.5 mM K_2_SO_4_ (+S) or 3 mM KCl (as a control) (-S) for 2 h. The FN plants were grown in the same medium and were harvested at the same time as were treated plants. At the end of the experiment, the shoots and roots were frozen in liquid nitrogen and stored at -80°C until further use. All experiments were performed in triplicate, and each replicate consisted of 80–100 seedlings.

### RNA Isolation and Quantitative Real-Time PCR (qPCR) Analysis

For gene expression analyses, the total RNA was isolated from the frozen roots and shoots using preheated (65°C) RNA extraction buffer [2% cetyl-trimethylammonium bromide (CTAB) (Sigma), 2% PVP-40 (Sigma), 100 mM Tris-HCl (pH 8), 30 mM EDTA, 2 M NaCl, and 2% mercaptoethanol] and then purified using mini columns (PureLink PCR Purification Kit, Invitrogen) in accordance with previously described protocols (Yaffe et al., 2012). First-strand cDNA was synthesized using 500 ng of total RNA and a Promega Go-Script Reverse Transcription System. Gene expression was measured by Touchdown qPCR assays (Zhang et al., 2015) with PowerUp SYBR Green Master Mix (Thermo Fisher Scientific) and a Stratagene Mx3000P qPCR System (Agilent). The raw fluorescence data were analyzed with Real-time PCR Miner software (Zhao and Fernald, 2005). Each qPCR sample (total volume of 20 μl) contained 10 ng of cDNA (10 ng) and each primer at 300 μM. The following PCR program was used: one cycle at 95°C for 3 min; four cycles at 95°C for 20 s followed by 66°C for 10 s, during which the temperature decreased by 3°C per cycle; and 40 cycles of 95°C for 30 s, 60°C for 30 s, and 72°C for 30 s. The gene-specific forward and reverse primers used are listed in **Supplementary Table S1**. The *clathrin adapter* gene (AT4G24550) was used as an internal control to quantify the relative mRNA levels (Czechowski et al., 2005).

### Microarray and Gene Co-expression Network Analyses

All previously published microarray experiments used in this work are listed in **Table 1**, and the raw data were downloaded from the Gene Expression Omnibus (GEO) database^1^. Several classic experiments (Hirai et al., 2003; Nikiforova et al., 2003; Maruyama-Nakashita et al., 2006) were not considered because of the lack of raw data in the public databases (GEO or ArrayExpress) or because of the microarray platform involved (only Affymetrix chips were considered). For each experiment, all the samples were normalized together using the robust multi-array average (RMA) method (Gautier et al., 2004) prior to differential expression analyses. To increase the power of the statistical analyses of the microarray data, we filtered out 50% of the genes that had lowest standard deviation, as previously recommended (Hackstadt and Hess, 2009). Differentially expressed genes were identified using empirical Bayes statistics implemented using the R package “limma” (Smyth, 2004). Those genes that met the following threshold criteria were considered significant: a minimum fold change (FC) of 2 and a false discovery rate (FDR) of less than 5%.

**Table 1:** Summary of microarray datasets and sample details.

Tissue	Growth stage	Growth condition	Medium	Age of plants at the beginning of the experiment	Age of plants at the end of the experiment	Reference
Root	Seedling	Agar plates	**MGRL medium:** MgSO_4_ was replaced by an equal molar concentration of MgCl_2_ in -S medium	10-day-old	11-day-old	Maruyama-Nakashita et al., 2005
Root	Seedling	Agar plates	**MS medium:** Sulfate salts in MS were replaced with equivalent chloride salts in -S medium	5-day-old	8-day-old	Iyer-Pascuzzi et al., 2011
Whole seedling	Seedling	Hydroponic	**Custom medium:** Macronutrients: 2 mM KNO_3_, 1 mM NH_4_NO_3_, 3 mM KH_2_PO_4_, 4 mM CaCl_2_, 1 mM MgSO_4_, 2 mM K_2_SO_4_, Micronutrients: 40 μM Na_2_FeEDTA, 60 μM H_3_BO_3_, 14 μM MnSO_4_, 1 μM ZnSO_4_, 0.6 μM CuSO_4_, 0.4 μM NiCl_2_, 0.3 μM HMoO_4_, 20 nM CoCl_2_	9-day-old	11-day-old	Bielecka et al., 2015
Root	Adult	Hydroponic	**Hoagland solution (half-strength):** MgSO_4_ was replaced by an equal molar concentration of MgCl_2_ in -S medium	14-day-old	49-day-old	Forieri et al., 2017
Root	Seedling	Agar plates	**MGRL medium:** MgSO_4_ was replaced by an equal molar concentration of MgCl_2_ in -S medium	0-day-old	10-day-old	Aarabi et al., 2016

To focus on consistent sulfate-responsive genes, we selected genes that were significantly regulated by sulfate in at least two different experiments. We then calculated the Pearson correlation coefficients between each gene pair across all selected experiments using the R package rsgcc (Ma and Wang, 2012). For network construction, an absolute correlation threshold of 0.81 was selected based on the best fit of the scale-free topology, as described previously (Romero-Campero et al., 2016). The gene co-expression network was visualized using Cytoscape v3.4 (Shannon et al., 2003), and the network topology parameters were calculated using the NetworkAnalyzer plugin (Doncheva et al., 2012). Co-expression modules were identified using Dynamic Tree Cut software (Langfelder et al., 2008).

### Functional Enrichment Analyses

Gene ontology (GO) enrichment analyses were performed using BiNGO software (Maere et al., 2005). Hypergeometric tests with an FDR of 5% as a cutoff were used to select significantly enriched GO terms. REVIGO software (Supek et al., 2011) was then used to reduce the redundancy between GO terms. In addition, we filtered and removed the general GO terms (those containing more than 5% of *A. thaliana* genes) to focus on the more specific terms.

## Results

### Meta-Transcriptomic Analysis Uncovers a New Core Set of Genes Involved in the Sulfate Response in *A. thaliana*

Several transcriptomic analyses of the response of *A. thaliana* plants to S starvation have been reported in the past several years (Maruyama-Nakashita et al., 2005; Iyer-Pascuzzi et al., 2011; Bielecka et al., 2015; Aarabi et al., 2016; Forieri et al., 2017). Additionally, microarray data from sulfate treatments after S-starvation periods have also been published (Maruyama-Nakashita et al., 2005; Bielecka et al., 2015). Therefore, a good opportunity exists to perform an integrated analysis of the sulfate transcriptomic data to answer relevant questions related to sulfate assimilation, such as “Is there a group of conserved genes involved in the response to sulfate availability?,” “What are the main biological functions involved in this response?,” and “What are the potential transcriptional regulatory factors of the sulfate response?”

**FIGURE 1:**
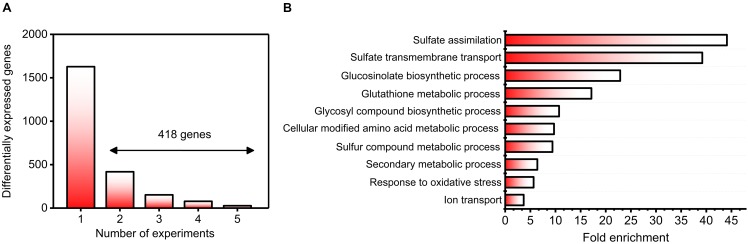
Identification of robust genes and biological functions involved in the sulfate response. **(A)** Histogram of 2046 significantly responding sulfate-responsive genes (FDR < 5%) distributed according to the number of experiments in which they were regulated. Those genes regulated by sulfate in at least two different experiments (418) were selected for further analyses. **(B)** The most enriched biological processes associated with genes regulated by sulfate availability. The GO over-representation analysis was carried out using 418 genes regulated by sulfate in at least two different experiments, and a 5% FDR cutoff was used. The top 10 GO terms are shown ranked in accordance with their enrichment level.

As a first step toward answering these questions, we conducted a detailed search for sulfate experiments in the GEO and ArrayExpress databases. Specifically, we selected experiments using the same microarray platform (Affymetrix) to avoid noise related to mixing different technologies, and we considered only samples from wild-type plants. Moreover, the microarray experiments in which raw data were not publicly available were discarded. In accordance with these criteria, five different datasets were found in public databases; these datasets were generated from experiments carried out in different laboratories (**Table 1**). Notably, three of these datasets were generated from time-course experiments that assessed the sulfate response, and there was a wide variety of sulfate concentrations, different growth systems (hydroponics or plates) and different tissues (roots and whole plants). In total, 52 microarrays were normalized by the RMA method (Gautier et al., 2004), and we performed a differential expression analysis between +S and -S samples from each experiment. To focus on the relevant S-responsive genes, we considered a gene to be differentially expressed when its FDR was less than 5% and its FC was a minimum of two. Using these criteria, we identified 2046 genes that significantly responded to changes in sulfate availability (**Figure 1A** and **Supplementary Table S2**). Only 20.43% of these differentially expressed genes were shared between at least two different experiments (418 genes, **Figure 1A** and **Supplementary Table S2**). Similar results have been reported for other nutrients and environmental factors, such as nitrate, carbon, or light conditions (Krouk et al., 2009; Canales et al., 2014). Among the most enriched biological functions associated with these 418 genes are sulfate assimilation, sulfate transport, GSL biosynthesis, and glutathione metabolism (**Figure 1B**). All these functions are directly related to sulfate assimilation (Takahashi et al., 2011; Kopriva et al., 2015), which is indicative of the high specificity of the genes identified in our analysis. On the other hand, the most enriched GO terms for molecular functions were “sulfate transmembrane transporter activity” (38-fold enrichment) and “glutathione transferase activity” (17-fold enrichment). The most enriched GO terms among the cellular components were “integral component of plasma membrane” (sevenfold enrichment) and “cell wall” (twofold enrichment). Taken together, these results indicate that the genes identified in our meta-analysis are highly associated with sulfate transport and metabolism.

**FIGURE 2:**
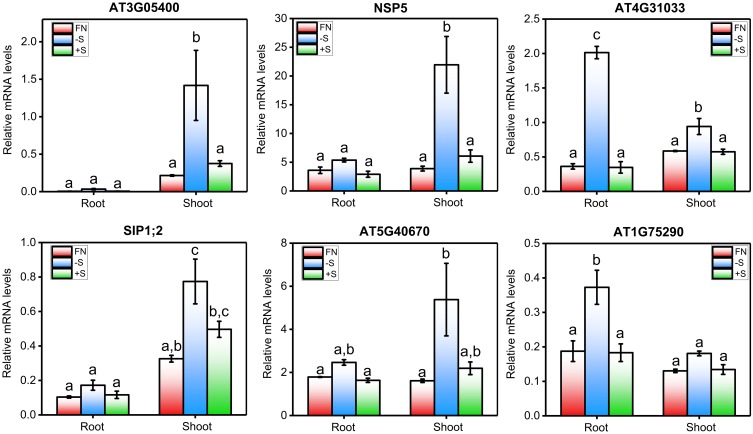
qPCR-analyzed expression profiles of highly consistent genes whose function in the sulfate response is unknown. mRNA levels of six genes (*AT3G05400*, *NSP5*, *AT4G31033*, *SIP1;2, AT5G40670*, and *AT1G75290*) selected from the meta-analysis of the sulfate transcriptome data were analyzed by qPCR using the *clathrin adapter* gene (AT4G24550) as a normalization reference gene. Briefly, 5-day-old seedlings were transferred to liquid MS medium that was supplemented with sulfate (FN, 1.5 mM S) or not, after which the seedlings were allowed to grow for 2 days. At dawn on the 7^th^ day, the plants were treated with potassium sulfate (+S, 1.5 mM) or potassium chloride (–S, 3 mM). The mRNA levels were measured 2 h after treatment using the total RNA extracted from root and shoot samples. The values plotted correspond to the means of three independent experiments ± standard errors of the mean; 80 to 100 seedlings were used per replicate. The means with different lowercase letters are significantly different, *p* < 0.05 (one-way ANOVA and Tukey’s test).

Interestingly, we found 27 genes that were differentially expressed in all the sulfate experiments analyzed (**Table 2**). Most (85.2%) of these genes were positively regulated by S starvation. Among this group, the genes *ATSDI1*, *BGLU28*, *AT5G26220*, and *LSU1* showed the greatest average induction in response to S starvation (>15-fold), and these genes have been used as markers of sulfate deficiency in several studies (Dan et al., 2006; Zhang et al., 2014; Aarabi et al., 2016). More than half of the genes in this group, such as *APR3* (Setya et al., 1996), *BCAT4* (Lächler et al., 2015), and *SULTR4;2* (Kataoka et al., 2004b), have a domain related to enzymatic activity or sulfate transport, suggesting that genes encoding transporters and enzymes related to sulfate assimilation are consistently transcriptionally regulated by sulfate availability. In addition, we identified a set of genes whose functions in the sulfate response are unknown, including *AT3G05400*, *AT4G31330*, *AT5G40670*, and *AT3G56200*, and that belong to the same group of consistent genes (≈45%, **Table 2**). To validate the results of this meta-analysis, we performed sulfate resupply experiments involving 7-day-old *Arabidopsis* seedlings that were previously subjected to 48 h of S starvation, as described in Section “Materials and Methods.” We collected root and shoot samples 2 h after sulfate treatment, and the mRNA levels of six genes that were representative examples of unknown and consistent sulfate-responsive genes were analyzed by qPCR (**Figure 2**). As shown in **Figure 2**, the genes *AT3G05400*, *NSP5*, *AT4G31330*, *SIP1;2*, *AT5G40670*, and *AT1G75290* were significantly induced at 48 h after S starvation. Similar results were also reported for the five experiments selected for our meta-analysis. Compared with the S-starvation treatment, the sulfate resupply treatment caused a significant decrease in the mRNA levels of these genes, with the exception of *SIP1;2*. This result indicates that *AT3G05400*, *NSP5*, *AT4G31330*, *AT5G40670*, and *AT1G75290* rapidly respond to changes in sulfate availability. Interestingly, we also found that the response of these genes to S starvation is tissue-dependent (**Figure 2**). For instance, *AT4G31330* showed a clear response to S starvation in the root tissue, whereas *AT3G05400* was specifically induced in the shoots, which suggests a different physiological role of these unknown genes in sulfate metabolism. In addition, six marker genes that respond to S were also analyzed by qPCR, and the expression profiles of these classic marker genes indicated that effective S treatments were performed under our specific experimental conditions (**Supplementary Figure S1**).

**Table 2:** Set of genes that significantly responded to sulfate in all the experiments analyzed (FDR < 5% and log_2_|FC| > 1).

ID	ALIAS	Description	log_2_FC (+S/-S)	Biological function
AT5G48850	ATSDI1	Tetratricopeptide repeat (TPR)-like superfamily protein	-4.65	Control of glucosionolate biosynthesis (Aarabi et al., 2016)
AT2G44460	BGLU28	Beta glucosidase 28	-4.31	Unknown, probably related with glucosinolate catabolism (Zhang et al., 2014)
AT5G26220	AT5G26220	ChaC-like family protein	-4.19	Glutathione catabolism (Paulose et al., 2013)
AT3G49580	LSU1	Response to low sulfur 1	-4.15	Unknown (Sirko et al., 2015)
AT5G24660	LSU2	Response to low sulfur 2	-3.32	Unknown (Sirko et al., 2015)
AT3G60140	DIN2	Glycosyl hydrolase superfamily protein	-2.77	Unknown
AT3G05400	AT3G05400	Major facilitator superfamily protein	-2.33	Unknown
AT4G31330	AT4G31330	Transmembrane protein	-2.30	Unknown
AT3G08860	PYD4	Pyrimidine 4	-2.16	Pyrimidine catabolism (Zrenner et al., 2009)
AT1G36370	SHM7	Serine hydroxymethyltransferase 7	-2.14	S Homeostasis (Huang et al., 2016)
AT1G04770	AT1G04770	Tetratricopeptide repeat (TPR)-like superfamily protein	-2.11	Control of glucosionolate biosynthesis (Aarabi et al., 2016)
AT4G21990	APR3	APS reductase 3	-1.96	S Assimilation (Setya et al., 1996)
AT5G10180	SULTR2;1	Sulfate transporter 2;1	-1.89	S Transport (Takahashi et al., 2000)
AT1G75290	AT1G75290	NAD(P)-binding Rossmann-fold superfamily protein	-1.88	Unknown
AT3G12520	SULTR4;2	Sulfate transporter 4;2	-1.77	S Transport (Kataoka et al., 2004b)
AT4G08620	SULTR1;1	Sulfate transporter 1;1	-1.66	S Transport (Takahashi et al., 2000)
AT3G56200	AT3G56200	Transmembrane amino acid transporter family protein	-1.46	Unknown
AT5G48180	NSP5	Nitrile specifier protein 5	-1.27	Unknown, probably related with glucosinolate catabolism (Kong et al., 2012)
AT5G40670	AT5G40670	PQ-Loop repeat family protein/transmembrane family protein	-1.04	Unknown
AT5G23050	AAE17	Acyl-activating enzyme 17	-1.01	Unknown
AT5G18290	SIP1;2	Aquaporin-like superfamily protein	-0.98	Water transport (Ishikawa et al., 2005)
AT3G27150	AT3G27150	Galactose oxidase/kelch repeat superfamily protein	-0.97	Unknown
AT2G25450	AT2G25450	2-Oxoglutarate (2OG) and Fe(II)-dependent oxygenase	0.74	Unknown
AT4G25835	AT4G25835	P-Loop containing nucleoside triphosphate hydrolases	0.86	Unknown
AT1G78370	GSTU20	Glutathione *S*-transferase TAU 20	0.90	Gluthatione metabolism (Wagner et al., 2002)
AT2G22330	CYP79B3	Cytochrome P450, family 79, subfamily B, polypeptide 3	1.54	Glucosinolate biosynthesis (Mostafa et al., 2016)
AT3G19710	BCAT4	Branched-chain aminotransferase4	1.59	Glucosinolate biosynthesis (Schuster et al., 2006)

### GO Analysis of Robust Sulfate-Responsive Genes Reveals the Biological Processes Involved in the Sulfate Response

**FIGURE 3:**
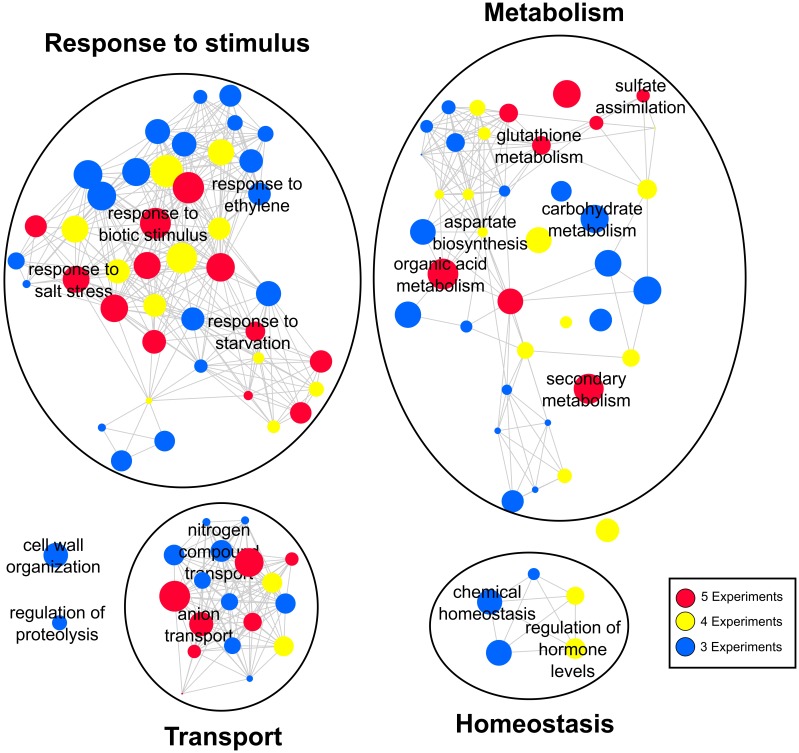
Gene ontology (GO) network of the most frequent biological functions regulated by sulfate. GO terms are linked by edges in the graph according to their semantic similarity (Supek et al., 2011). The bubble size indicates the percentage of genes associated with the corresponding GO term, and the color indicates the degree of consistency of the corresponding GO term.

Determination of the most consistent genes in the sulfate response immediately raises the following question: what are the most consistent biological functions? To answer this question, we subjected each sulfate experiment to a functional enrichment analysis using BiNGO software (Maere et al., 2005), after which we ranked the over-represented GO terms according to their consistency across the five experiments analyzed. REVIGO software (Supek et al., 2011) was used to remove redundant GO terms and was also used to construct a network consisting of the most consistent biological processes (**Figure 3**). As such, we identified four major functional groups: response to stimulus, metabolism, transport, and homeostasis (**Figure 3**). The GO terms related to ion transport, sulfate metabolism and response to stress were the most consistent biological functions that were over-represented in all the experiments analyzed (red circles, **Figure 3**). Moreover, we found several consistent GO terms related to biological processes that have been studied little in the context of the sulfate response, including cell wall organization, regulation of proteolysis, carbohydrate metabolism, and nitrogen compound transport. Consistent with these results, a strong interaction between the sulfate and nitrate assimilation pathways has been reported (Kopriva and Rennenberg, 2004). Interestingly, our meta-analysis identified genes encoding nitrate transporters [NTR2.1 (Wang and Crawford, 1996) and NTR3.1 (Okamoto et al., 2006)] and important nitrogen metabolism enzymes, such as nitrate reductase [NIA1 and NIA2 (Cheng et al., 1991)] and asparagine synthetase [ASN2 (Lam et al., 1998)], which were differentially expressed in accordance with sulfate availability in at least two different experiments (**Supplementary Table S2**). Moreover, we have found that overlaps between sulfate-responsive genes identified in this work and nitrate-responsive genes reported previously (Canales et al., 2014) are higher than expected due to chance (*p*-value < 0.001, Genesect Virtual Plant 1.3; Katari et al., 2010). Specifically, 169 genes are shared between a set of 1021 nitrate-responsive genes and a set of 418 sulfate-responsive genes (**Supplementary Figure S2**). Interestingly, among the most over-represented biological functions of these shared genes, we found “response to stimulus” and “secondary metabolism” GO terms; these shared genes include multiple glutathione metabolism genes, including *GSTU20*, *GSTU22*, *GSTU24*, and *GSTU25* (Wagner et al., 2002).

### Gene Co-expression Network Analysis Predicts New Candidate Genes and Functional Modules Involved in the Sulfate Response

**FIGURE 4:**
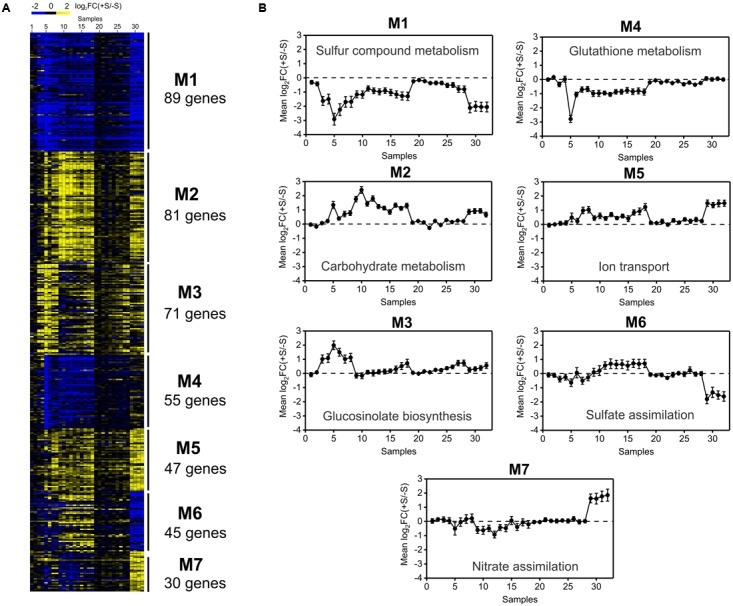
Hierarchical clustering of transcriptomic data from sulfate-availability experiments uncovers functional modules involved in the sulfate response. **(A)** Heatmap of 418 sulfate-responsive genes selected in the meta-analysis. Gene co-expression modules identified using Dynamic Tree Cut software are indicated on the right-hand side of the heatmap. Genes up-regulated by sulfate are indicated in yellow, and genes down-regulated by sulfate are shown in blue. **(B)** Average expression profiling of each co-expression module across the different samples analyzed in the meta-analysis. Samples 1 to 6 correspond to the experiment of Bielecka et al. (2015), samples 7 and 8 correspond to the experiment of Aarabi et al. (2016), samples 9 to 18 correspond to the dataset of Iyer-Pascuzzi et al. (2011), samples 19 to 28 correspond to the dataset of Maruyama-Nakashita et al. (2005) and samples 29 to 32 correspond to the experiment of Forieri et al. (2017). The GEO accession number for each sample is indicated in **Supplementary Table S4**. The most over-represented GO term for the biological processes is indicated within each module.

We hypothesized that an unsupervised gene co-expression network analysis of the sulfate transcriptomic data will provide new insight into the regulation of sulfate metabolism and will help prioritize candidate genes for future functional analyses. To reduce noise in our dataset for co-expression analyses, we focused on genes that were differentially expressed in at least two different experiments (418 genes, **Figure 1A**). First, prior to network construction, we performed unsupervised hierarchical clustering using Dynamic Tree Cut software (Langfelder et al., 2008) to identify gene co-expression modules associated with the sulfate response. As such, we identified seven major modules, which were sorted and named according their size from 1 to 7. As shown in **Figure 4**, modules M1 and M4 show a clear negative response to sulfate, whereas M2, M3, and M5 show a general positive response to sulfate availability. The functional enrichment analysis revealed that the modules with a negative response to sulfate (M1 and M4) had over-represented biological functions related to sulfate assimilation and glutathione metabolism (**Figure 4**). In contrast, positive responsive modules, such as M2, M3 and M5, had over-represented biological functions related to secondary S metabolism, ion transport and carbon responses (**Figure 4**). This result indicates that S-starvation signals activate the expression of genes related to sulfate assimilation and repress genes related to secondary S metabolism, as reported previously (Hirai et al., 2005; Kopriva et al., 2012), which reflects the accuracy of our predictions. Notably, our analysis extends this regulatory mechanism to other biological processes such as the carbon response (M3) and transport of other molecules such as nitrate or amino acids (M5). On the other hand, genes of modules M6 and M7 did not show a consistent response to S, indicating that other factors interact with the S response to control the expression of these genes. These modules, M6 and M7, included genes related to nitrate and sulfate metabolism, such as *NIA1*, *NIA2*, *APR2*, and *ATSERAT3;1*.

**FIGURE 5:**
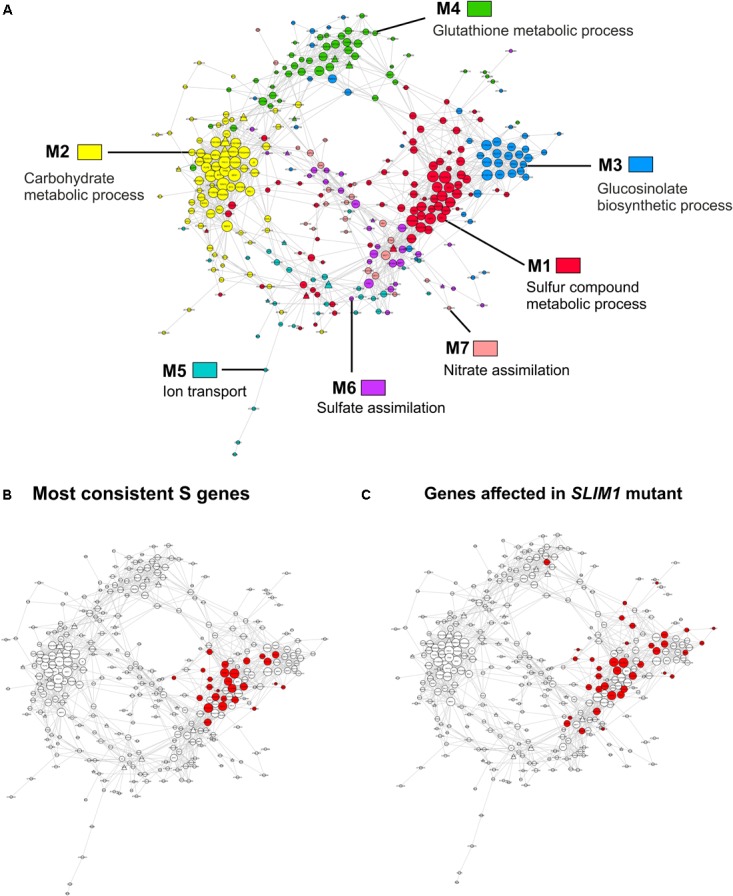
Sulfate-responsive gene co-expression network. **(A)** Colors are used to distinguish the genes from each network module. The most over-represented GO terms for the biological process are indicated in each module. The shape of each specific node is related to TFs (triangle) or targets (circle) and the size depends on the number of connections of each node in such a way that nodes with higher size are the most connected genes in the network. **(B)** The most consistent sulfate-responsive genes in the co-expression network. The red nodes indicate genes that were regulated by sulfate availability in the five analyzed experiments. **(C)** Mapping of the genes that were significantly affected in *slim1* mutants according to previous microarray analyses (Maruyama-Nakashita et al., 2006).

To better understand the functional relationships between gene co-expression modules and to identify new candidate genes related to the sulfate response, we performed a gene co-expression network analysis. As such, using the R package rsgcc, we determined the Pearson’s correlation index of each pair of sulfate-responsive genes (Ma and Wang, 2012). We then selected a Pearson correlation threshold (0.81) based on the best fit of the scale-free model (**Supplementary Figure S3**), as previously described (Romero-Campero et al., 2016). Accordingly, we obtained a gene co-expression network with 341 nodes and 1811 connections as shown in **Figure 5A**. Gene co-expression modules (M1–M7) are marked with different colors to distinguish the different expression patterns and functional groups present in the network.

With 86 genes, M1 is the largest module in the sulfate-responsive gene co-expression network. Moreover, M1 contains most of the genes that were consistently detected in our meta-analysis of the sulfate response (**Figure 5B**, 21 of 23 genes), suggesting that this module plays a key role in the sulfate response. In addition, most of these genes were affected in plants with a mutant form of *slim1* (**Figure 5C**), a key TF involved in the control of the sulfate assimilation pathway (Maruyama-Nakashita et al., 2006). The top GO categories in this module are S compound metabolic processes (GO:0006790, adjusted *p*-value = 8.22E-12) and S compound transport (GO:0072348, adjusted *p*-value = 8.22E-12). In network analyses, a common way to prioritize candidate genes is by the number of connections (van Dam et al., 2017), which is also known as the degree of a gene. Thus, NetworkAnalyzer was used to determine the degree of each gene from this module (Doncheva et al., 2012). The genes of high degree in this module were *SULTR4;2* [a vacuolar sulfate transporter (Kataoka et al., 2004b)], *AT3G56200* (a putative amino acid transporter) and *SHM7* [serine hydroxymethyltransferase 7 (Huang et al., 2016)], each of which had 34, 32, and 31 connections, respectively (**Table 3**).

**Table 3:** Ranking of the 10 most connected genes in the sulfate co-expression network.

ID	ALIAS	Description	Degree	Module
AT5G20250	DIN10	Raffinose synthase family protein	36	2
AT4G35770	SEN1	Rhodanese/cell cycle control phosphatase superfamily protein	36	2
AT3G12520	SULTR4;2	Sulfate transporter 4;2	34	1
AT5G24490	AT5G24490	30S Ribosomal protein	34	2
AT2G18700	TPS11	Trehalose phosphatase/synthase 11	34	2
AT3G56200	AT3G56200	Transmembrane amino acid transporter family protein	32	1
AT1G80440	AT1G80440	Galactose oxidase/kelch repeat superfamily protein	32	2
AT1G36370	SHM7	Serine hydroxymethyltransferase 7	31	1
AT5G49450	bZIP1	Basic leucine-zipper 1	31	2
AT3G29240	AT3G29240	PPR containing protein (DUF179)	31	2

M3 is the module most connected to M1; a total of 75 connections were present between the genes of the two modules (**Figure 5A**). The top GO categories in M3 were GSL biosynthetic process (GO: 0019761, adjusted *p*-value = 3.78E-27) and glycosyl compound biosynthetic process (GO: 1901659, adjusted *p*-value = 7.85E-22). Several well-known enzymes involved in the GSL biosynthetic pathway, including CYP83A1, IMD3, BCAT4, and MAM1 (Sønderby et al., 2010), are among the most connected genes in this module. As mentioned above, the expression patterns of the genes in modules M1 and M3 exhibit contrasting responses to sulfate (**Figure 4**), indicating that these genes are inversely regulated by S-starvation signals (Falk et al., 2007; Hirai et al., 2007; Davidian and Kopriva, 2010).

**FIGURE 6:**
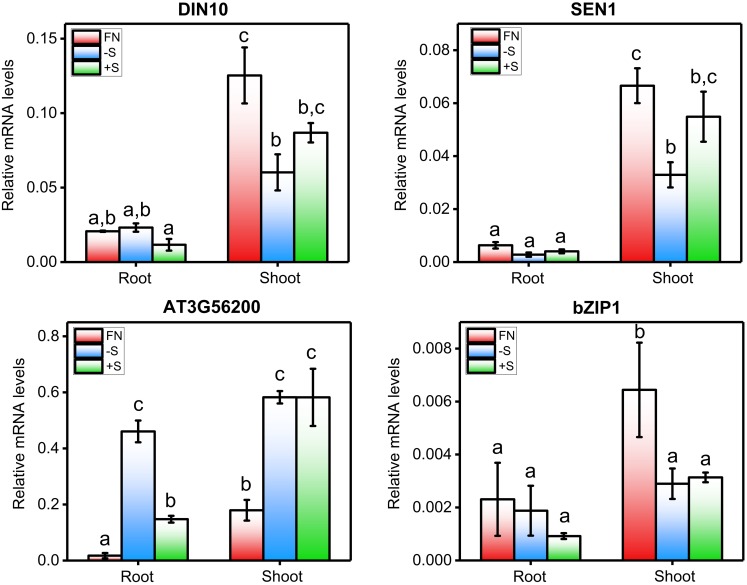
qPCR-analyzed expression profiles of hub genes identified in the sulfate co-expression network. The mRNA levels of *DIN10*, *SEN1*, *AT3G56200*, and *bZIP1* were determined by qPCR in three different samples: FN (full nutrient-treated samples), +S (sulfate-treated samples) and –S (sulfate-starved samples). For more details regarding this experiment, see the legend in **Figure 2**. The values plotted correspond to the means of three independent experiments ± standard errors of the mean; 80 to 100 seedlings were used per replicate. The means with different lowercase letters are significantly different, *p* < 0.05 (one-way ANOVA and Tukey’s test).

The second largest module in the sulfate co-expression network is M2, which contains 81 genes. In this case, the carbohydrate metabolic process (GO: 0005975, adjusted *p*-value = 4.26E-03) and the response to carbohydrate (GO: 0009743, adjusted *p*-value = 4.26E-03) were the most over-represented biological processes. In fact, several of the most connected genes of this module are related to these biological functions, including *DIN10* (Fujiki et al., 2001), *bZIP1* (Usadel et al., 2008) and *TPS8* (Ramon et al., 2009), each of which has 36, 31, and 30 connections, respectively (**Supplementary Table S3**). To verify the expression of the selected genes, using the same conditions as mentioned previously, qPCR expression analyses were performed. As shown in **Figure 6**, the expression levels of *DIN10* and *bZIP1* in aerial tissues were down-regulated in response to sulfate deficiency and did not recover after 2 h of sulfate treatment. In addition, we also analyzed the expression of a gene related to tRNA processing, *SEN1* (Akama et al., 2000), which, together with *DIN10*, is the most connected gene in the sulfate co-expression network (**Table 3**). Interestingly, the RNA levels of the *SEN1* gene were significantly higher in sulfate-containing samples (FN and +S) than in sulfate-starved samples (-S), and similar responses were observed for *DIN10* and *bZIP1* (**Figure 6**). Overall, the results of the qPCR analyses confirm the expression patterns obtained by the microarray data (**Figure 4B**, M2).

The most connected module to M2 is M4, which is related to glutathione metabolism (GO:0006749, adjusted *p*-value = 1.31E-07). As shown in **Figure 4**, the average expression profile of module M4 contrasts with that of module M2, indicating that several connected genes of the two modules are negatively correlated with each other. Other biological processes over-represented in M4 included the flavonoid metabolic process (GO:0009812, adjusted *p*-value = 2.66E-07) and cellular-modified amino acid metabolic process, indicating that this module is enriched in genes related to secondary metabolism.

### Temporal Dynamics of the Network Reveal a Complex Response to S Starvation

**FIGURE 7:**
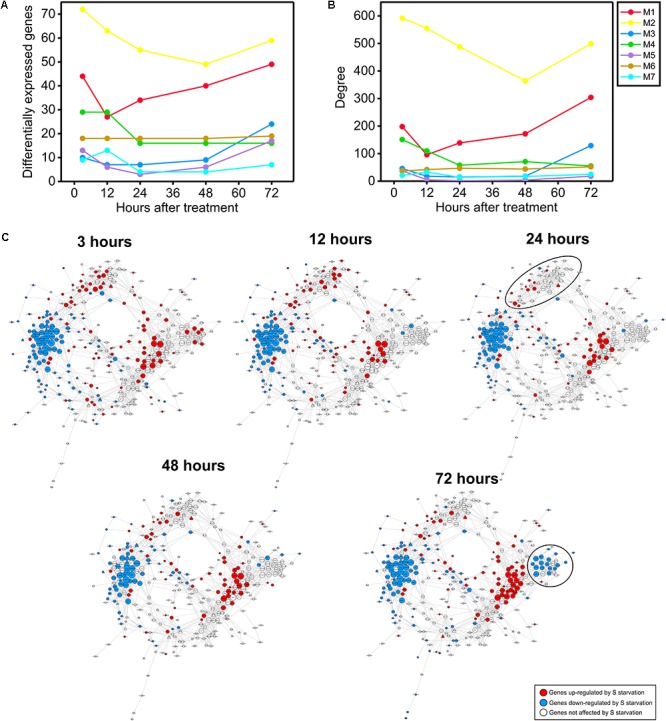
Temporal dynamics of the sulfate co-expression network. **(A)** Temporal dynamics of the numbers of genes differentially expressed in response to sulfate starvation in a previous experiment (Iyer-Pascuzzi et al., 2011). The color code indicates the co-expression module to which each gene belongs according to the sulfate co-expression network presented in **Figure 5**. **(B)** Temporal dynamics of the degree of each co-expression module according to the differentially expressed genes in the previously mentioned experiment (Iyer-Pascuzzi et al., 2011). The color code indicates the co-expression module. **(C)** Mapping of the differentially expressed genes in response to sulfate starvation at five different time points (3, 12, 24, 48, and 72 h). The red and blue colors indicate significant up- or down-regulation, respectively, in response to sulfate starvation (adjusted *p*-value < 0.05 and log_2_|FC| > 1 or < –1). The microarray data were obtained from the experiment GSE30098 of GEO (Iyer-Pascuzzi et al., 2011). The shape of each specific node is related to TFs (triangle) or targets (circle), the size depends on the number of connections of each node in such a way that nodes with higher size are the most connected genes in the network.

To further investigate the dynamics of the sulfate response, we performed detailed expression and network analyses of *Arabidopsis* plants subjected to S starvation. For this purpose, we selected the available microarray dataset that contained the most differentially expressed genes and the most time points (Iyer-Pascuzzi et al., 2011). Specifically, 7-day-old plants were subjected to S starvation, and samples were collected at five different time points after this treatment (0, 3, 12, 24, 48, and 72 h). We first analyzed the number of differentially expressed genes identified in modules M1–M7 at the different time points after the starvation treatment (**Figure 7C**). In addition, we determined the node degree for the different modules, which served as an estimation of the complexity of the gene co-expression network at the different time points.

As shown in **Figure 7A**, most of the sulfate co-expression network modules exhibited a similar expression pattern; maximal values were reached at early (3–12 h) and late (60–72 h) time points after the starvation treatment. An exception to these general patterns were the genes of module M6; these genes exhibited stable expression patterns in response to S starvation across all time points. Similar results were obtained when we assessed the number of connections or the degree of each module (**Figure 7B**). In fact, most of the sulfate modules present higher degree values at early (3–12 h) and late (60–72 h) time points. Taken together these results suggest that the S-starvation response likely generates a biphasic response, which involves major changes in gene regulatory networks during both the early and late responses. A similar biphasic response to S-starvation has been reported after performing an integrative analysis of transcriptomic and metabolomic data at longer times (Nikiforova et al., 2004; Hoefgen and Nikiforova, 2007).

### Network Analysis Predicts That the Sulfate Response Is Regulated by a Limited Number of TFs, Including MYB, bZIP, and NF-YA

Regarding TFs, we found 17 genes annotated as “nucleic acid binding” (GO:0003676) in the sulfate co-expression network; these genes represented 4.5% of the genes with GO annotations. In contrast, in the entire *A. thaliana* genome, 14% of genes were annotated as “nucleic acid binding,” which is three times greater than the percentage of genes involved in the sulfate co-expression network. Genes with nucleic acid-binding domains were significantly underrepresented (adjusted *p*-value = 3.44E-06), suggesting that most of the TFs involved in the response to sulfate availability are not transcriptionally regulated. Since, we have applied a twofold of change filter for the selection of differentially expressed genes, it is possible that this filter has specifically reduced the number of TFs. The number of differentially expressed genes increased up to 2148 by removing the twofold expression filter. Despite this increase in the number of genes, the GO term “nucleic acid binding” (GO:0003676) was also significantly under-represented (adjusted *p*-value = 3.6667E-8), indicating that the twofold expression filter is not the reason of TFs under-representation.

**FIGURE 8:**
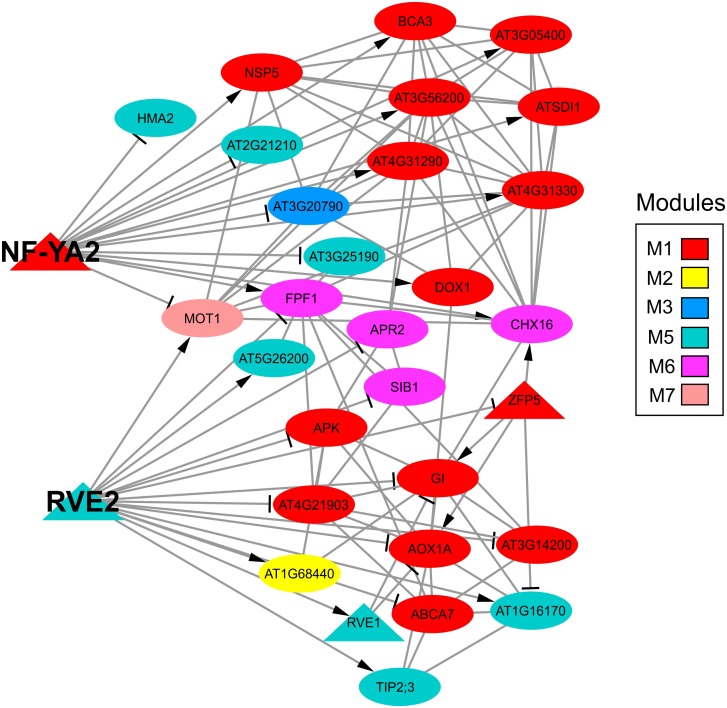
Subnetwork of two TFs (NF-YA2 and RVE2) identified as intermodular hub genes of the sulfate gene co-expression network. The color code indicates the co-expression module to which each gene belongs according to the sulfate co-expression network presented in **Figure 5**. The arrows or lines at the end of an edge indicate positive or negative correlations, respectively. The shape of each specific node is related to TFs (triangle) or targets (circle) and the size depends on the number of connections of each node in such a way that nodes with higher size are the most connected genes in the network.

Another possibility is that the sulfate-responsive TFs identified in this network have many targets. The five TFs with the highest degree were co-expressed together alongside a total of 66 different genes, which represent 19.35% of the sulfate network. Specifically, the TFs with the highest degree in the sulfate co-expression network were bZIP1 (Weltmeier et al., 2009), ATCTH (Pomeranz et al., 2010) and MYBL2 (Matsui et al., 2008), each of which has 31, 25, and 16 connections, respectively (**Supplementary Table S3**). These three TFs belong to module M2, and most of their connections are with genes from the same module, indicating that these TFs are specific to M2. After analyzing all the TFs in the sulfate co-expression network, we identified two TFs connected to multiple co-expression modules, suggesting that these TFs could be important for the coordination of different functional responses to sulfate. These TFs include RVE2 [AT5G37260 (Zhang et al., 2007)] and NF-YA2 [AT3G05690 (Leyva-González et al., 2012)] and are connected to specific genes of five different co-expression modules (**Figure 8**). In fact, RVE2 has 16 connections that are distributed among M1 (6), M2 (1), M5 (5), M6 (3), and M7 (1). NF-YA2 has 15 connections with genes in modules M1 (9), M3 (1), M5 (3), M6 (2), and M7 (1); thus, these two TFs are connected to the same functional modules, with the exceptions of M2 and M3. However, RVE2 and NF-YA2 share only two target genes (*MOT1* and *FPF1*), suggesting that RVE2 and NF-YA2 act complementarily to regulate different genes of the same modules.

## Discussion

Although several transcriptomic studies have been performed in the past decade, an integrated view of the transcriptional regulation of the sulfate response is still lacking. The identification of relevant genes in the response to an environmental stimulus is an important step in understanding the molecular mechanisms involved in this process. The analysis of multiple transcriptomic experiments focuses on the same stimulus is often used to achieve this goal (Tseng et al., 2012). This type of analysis is based on the logical principle that the expression of essential genes controlling the response to a stimulus is independent of other factors, such as developmental stage or plant growth conditions. Moreover, meta-analyses are useful for the identification of new genes and biological processes related to a stimulus of interest, such as nutrient availability (Canales et al., 2014) or a phytohormone (Bhargava et al., 2013). For instance, a meta-transcriptomic analysis of the nitrate response revealed that root hair development was one of the most consistent biological functions in that response (Canales et al., 2014). This result was based on bioinformatic analyses that have recently been experimentally validated in a report showing that the molecular mechanism involved in root hair development is induced by nitrate treatment (Canales et al., 2017). Following a similar strategy, we present a detailed meta-analysis of sulfate-specific microarray experiments; this meta-analysis enabled us to identify a highly consistent group of sulfate-responsive genes. Several well-known genes involved in sulfate transport and metabolism, such as *SULTR1;1*, *SULTR2;1*, *SULTR4;2* and *APR3*, are members of this group (Takahashi, 2010). Interestingly, most of these consistent genes were also highly co-expressed in sulfate-specific experiments and belong to module M1 of the sulfate co-expression network. Based on the biological functions associated with the genes in M1, these highly consistent genes in the sulfate response are likely strongly related to sulfate transport and metabolism.

However, M1 also contains several genes whose functions are unknown but that are related to sulfate metabolism, such as *AT3G56200*, *AT4G31330*, and *AT3G05400*. In the case of *AT3G56200*, the predicted open reading frame codes for a protein that consists of 435 amino acids and contains a conserved transmembrane domain (pfam01490) present in several amino acid transporters including amino butyric acid (GABA) transporters (Edwards et al., 1997), proline transporters and amino acid permeases (Marchler-Bauer et al., 2017). In accordance with SUBA4 prediction and consistent with the presence of this transmembrane domain, the subcellular localization of *AT3G56200* is the plasma membrane (Hooper et al., 2017). The qPCR expression analyses revealed that *AT3G56200* was significantly induced in the roots and shoots of *Arabidopsis* under S deficiency conditions. Interestingly, the resupply of this nutrient 2 h later reduced the expression of this gene only in the roots, possibly because it takes more time for sulfate or its derivatives to be transported to the aerial portions. Considering these data, the *AT3G56200* gene might be involved in the transport of amino acids derived from sulfate assimilation, such as Cys, methionine, or serine. Interestingly, this gene is highly co-expressed together alongside genes related to sulfate assimilation, including SULTR4;1, APS1, and APS3 [ATTED-II (Obayashi et al., 2017)]. However, we cannot rule out the possibility that the product of this gene, acting as co-transporter, plays an indirect role in the amino acid transport or is involved in the transport of other S-containing compounds. Owing to its root-specific response to S, the *AT4G31330* gene, whose function is unknown, stands out in this group; this gene codes for a putative protein that consists of 239 amino acids and, in accordance with SUBA4 prediction, localizes to the plasma membrane (Hooper et al., 2017). Interestingly, the *AT4G31330* gene is highly co-expressed together alongside a group of genes coding for several enzymes involved in amino acid metabolism, such as aspartate kinase 3 (Yoshioka et al., 2001), methionine sulfoxide reductase B5 (Vieira Dos Santos et al., 2005) and ATSERAT3;1, suggesting that this gene is also related to amino acid transport. Moreover, information within a general co-expression database [ATTED-II (Obayashi et al., 2017)] suggests that *AT4G31330* is highly co-expressed together alongside the genes coding for several members of the nodulin MtN21-like transporter family, including *UMAMIT12*, *UMAMIT20* and *UMAMIT28*, which are usually involved in amino acid export (Muller et al., 2015). Importantly, the genes involved in the transport of S-containing amino acids are currently unknown (Gigolashvili and Kopriva, 2014). Our analyses indicated that the *AT3G56200* and *AT4G31330* genes might play a relevant role in this important biological process for plants to adapt to changes in S availability.

Another interesting finding from our analysis is that changes in sulfate availability regulate genes involved in nitrate metabolism. Regulatory interactions of sulfate and nitrate reduction have been reported in several plant systems (Lee et al., 2011). For example, the activity of nitrate reductase decreases upon S starvation in cultured tobacco cells (Reuveny et al., 1980). Moreover, it has been reported that S deficiency also affects the internal nitrate levels in *Arabidopsis* (Forieri et al., 2017), as nitrate metabolism is among the biological processes that were significantly down-regulated in a recent transcriptomic analysis (Forieri et al., 2017). In addition, carbon availability is an important factor that affects the expression of genes that are involved in nitrate and sulfate assimilation (Hesse et al., 2003; Gutiérrez et al., 2007). For example, glucose and sucrose treatments induced the expression of the important enzymes of sulfate metabolism, such as APR and sulfate transporters (Hesse et al., 2003; Maruyama-Nakashita et al., 2004). In the case of nitrogen, *asparagine synthetase 1* (*ASN1*) was one of the first reported genes showing dual regulation by nitrogen and carbon and is related to the role of asparagine, which represents an efficient compound for nitrogen transport (Lam et al., 1994). Several genes involved in the control of carbon/nitrogen (C/N) balance have subsequently been reported in *Arabidopsis*, such as bZIP1 (Obertello et al., 2010; Dietrich et al., 2011) and ATL31 (Sato et al., 2009). Interestingly, we found that S starvation reduces the expression levels of *ASN1* and *bZIP1*, suggesting that the C/N balance is affected by sulfate availability. Thus, it is possible that C/N balance is among the signals involved in the coordination of S and nitrogen metabolism. Indeed, the connections between nitrogen, carbon, and S metabolism involve two different regulated branches for the biosynthesis of Cys (Dong et al., 2017). One branch is affected by the limitation of C/N and is regulated by GCN2 protein kinase for the selective sensing of Cys precursors (Dong et al., 2017). However, by down-regulating glucose metabolism, S starvation is transduced to TOR (Dong et al., 2017). In addition to these novel results, our analysis suggests that the bZIP1 signaling pathway might also be involved in the coordination of carbon, S, and nitrogen metabolism.

Topological parameters of the presented gene co-expression network can be used to identify new candidate genes. This criterion is based on empirical evidence showing a positive correlation between the essentiality and connectivity of a gene (Jeong et al., 2001; Hahn and Kern, 2005; Carlson et al., 2006). In our case, the most connected genes in the network were *SEN1* and *DIN10*, both of whose functions in the sulfate response are unknown. These two genes belong to a group of dark-inducible (*DIN*) genes (Azumi and Watanabe, 1991), whose expression is strongly induced by low-carbon conditions, including darkness and senescence (Fujiki et al., 2001). Both the microarray data and qPCR analyses reveal that *SEN1* and *DIN10* are repressed by conditions of S deficiency. Other *DIN* genes, such as *ASN1*, *DIN2* and *DIN9*, responded similarly, suggesting that S starvation increases carbon availability. This hypothesis is supported by several metabolic studies that have shown significant increases in raffinose and sucrose levels upon S starvation (Nikiforova et al., 2005; Zhang et al., 2011); these increases might be due to the activation of GSL catabolism, which releases sugars and S metabolites (Maruyama-Nakashita, 2017). Other biological functions highlighted by our meta-analysis include cell wall organization and the regulation of proteolysis. Two extracellular polypeptides are related to the restructuring of cell walls during S starvation in *Chlamydomonas reinhardtii* for redistributing internal S-containing molecules (Takahashi et al., 2001). In accordance with this biological function, genes coding for apoplast-localized proteins were also over-represented in the sulfate-responsive genes detected in our meta-analysis. The modification of cell walls during S starvation might also be related to nutrient uptake, since the permeability of the plant cells depends largely on the composition of this extracellular barrier. S deficiency can increase the suberization of root endodermis within cell walls, and interestingly, plants with mutant forms of the sulfate transporters SULTR1;1 and SULTR1;2 displayed increased suberization (Barberon et al., 2016). We also identified several genes related to the regulation of proteolysis, a biological function that might be related to the remobilization of S from proteins. For instance, significant degradation of ribulose-1,5-bisphospate carboxylase occurs during S deprivation (Ferreira and Teixeira, 1992). This biological function might also be involved in the post-translational control of the S response. By using our meta-analysis, we identified two sulfate-responsive genes that are members of the *Arabidopsis* Tóxicos en Levadura (ATL) family (ATL8 and ATL15), a group of plant-specific RING-type ubiquitin ligases involved in ubiquitin-dependent protein catabolic processes (Aguilar-Hernández et al., 2011).

Transcription factors are important components involved in the control of gene regulatory networks; therefore, we analyzed this protein family in the sulfate co-expression network in high detail. Unexpectedly, we found that TFs were significantly underrepresented in the sulfate co-expression network. Importantly, only two TFs that are involved in the sulfate response and functionally characterized thus far (SLIM1 and HY5) are not regulated by this nutrient (Maruyama-Nakashita et al., 2006; Lee et al., 2011; Koprivova and Kopriva, 2014). Overall, these data support the idea that, in addition to transcriptional regulatory mechanisms, other post-transcriptional and post-translational mechanisms are important for plant sulfate responses. MYB28, a key TF involved in GSL biosynthesis, is repressed by interactions with SDI1 and SDI2 proteins, which in turn are induced by S starvation (Aarabi et al., 2016). These interacting proteins localize to the nucleus but lack the DNA-binding ability, indicating that SDIs proteins might represents an additional layer of regulation. To date, 59 miRNAs whose expression is affected by S deficiency have been described (Liang et al., 2015). However, only the family of miR-395 has been shown to mediate sulfate allocation and regulate the expression of important enzymes of sulfate assimilation, such as APS3/APS4 (Kawashima et al., 2009, 2011; Liang et al., 2010). Post-translational regulatory mechanisms controlling the S response are largely unknown, since no proteomic studies have been conducted in *Arabidopsis* seedlings or adult plants under S deficiency conditions.

Another reason that may explain the underrepresentation of TFs in the sulfate-responsive co-expression network is that some TFs have many targets. Our network analyses predicted that only five TFs control the expression of a large proportion of the sulfate-regulated target genes identified in this work (approximately 20% of the total set of sulfate-responsive genes). We highlight the case of the NF-YA2 and RVE2 TFs, since they are connected to genes within five different co-expression modules, suggesting that these TFs might be central regulators of the S response. NF-YA2 belongs to a family of evolutionarily conserved TFs present in nearly all eukaryotes (Zhao et al., 2017). NF-Y TFs are biologically active upon formation of a heterotrimeric complex composed of NF-YA, NF-YB, and NF-YC subunits (Nardini et al., 2013). NF-Y TFs are associated with the regulation of several developmental processes such as flowering and root system architecture (Petroni et al., 2012). NF-YA2 is preferentially expressed in leaves and shoot apical meristems, and overexpression of NF-YA2 increases leaf biomass (Zhang et al., 2017). Our analysis showed that NF-YA2 is positively correlated with the genes in modules M1 and M6, which are related to S metabolism and the response to S deficiency. Interestingly, NF-YA2 is regulated by other nutritional deficiencies such as phosphate starvation (Woo et al., 2012), suggesting a general role of this TF in the nutrient starvation response. On the other hand, RVE2 is also co-expressed together alongside genes in the same modules with which NF-YA2 is associated but in an inverse manner. Our microarray analysis revealed that RVE2 is repressed by S starvation, whereas NF-YA2 is induced by this signal, suggesting these TFs act complementarily to coordinate the response to sulfate availability. RVE2 is a MYB-like TF and has a single MYB domain that belongs to the same family as that of CCA1 and LHY (Rawat et al., 2009), which are important TFs involved in the control of the circadian rhythm and flowering (Park et al., 2016). In fact, the expression levels of the central regulators of the circadian rhythm are affected by overexpression of RVE2 (Zhang et al., 2007). Recently, it has been reported that, by directly regulating gibberellin biosynthesis, RVE2, together with RVE1, regulates important developmental processes such as germination in *Arabidopsis* (Jiang et al., 2016); this finding suggests that S signaling pathways are also involved in the control of other developmental processes such as flowering and germination.

Based on our analysis of microarray data, we identified 2046 sulfate-responsive genes and the most robust biological functions associated with them in *Arabidopsis*. However, individual transcriptomic analyses of the sulfate response are unable to capture the broad realm of sulfate-responsive genes because these experiments are carried out under very specific experimental conditions (Hirai and Saito, 2004). Similar to other environmental factors, responses to changes in S availability depend on multiple factors, such as time of treatment, nutrient concentrations, and age of the plant or tissue (Hirai and Saito, 2004). We found that most of the sulfate-responsive genes are regulated under a particular experimental condition (**Figure 1A**). Accordingly, only 18.2% of genes reported by one of the pioneering microarray analyses (Hirai et al., 2003) are shared with those identified from our meta-analysis (**Supplementary Figure S4**). The low overlap with this experiment might be due to differences in the age of the plants used in that experiment (21 days old), as most of the samples used in our meta-analysis were from plants that were 7 to 13 days old. In contrast, the data obtained by Nikiforova et al. (2003) and Maruyama-Nakashita et al. (2006) showed greater overlap with the data in our meta-analysis: 33.7 and 59.5%, respectively. Interestingly, the age range of the plants analyzed in those experiments was similar to that of the plants used in our meta-analysis. Taken together, these results suggest that developmental stage might be an important factor that affects the transcriptional response to sulfate availability, which has been reported for the case of nitrate in maize (Plett et al., 2016).

Another possibility to explain the limited overlap between individual experiments observed in this study is that the consistency of nutrient responses is markedly higher at the biological function level than at the gene level (Canales et al., 2014). We found that the median overlap between the over-represented biological functions of any combination of the five experiments analyzed in this work was 52%, whereas at the gene level, the median overlap was 23%. The overlap between our meta-analysis and the pioneering works reported by Hirai et al. (2003), Nikiforova et al. (2003), and Maruyama-Nakashita et al. (2006) is also consistently higher at biological function level than at the gene level (**Supplementary Figure S4**). This result indicates that the same biological function, e.g., sulfate transport, can be carried out by different genes depending on the environmental and developmental context.

## Conclusion

Our work also highlights the role of the TFs bZIP, MYB, and NF-YA in the regulation of important functions related to sulfate transport and signaling. NF-YA TFs have not yet been characterized in the framework of sulfate responses. Moreover, we identified candidate genes involved in the transport of amino acids derived from sulfate assimilation. Functional studies of these new candidate genes should improve the understanding of the regulatory mechanisms underlying sulfate responses in crop species and model plant species such as *Arabidopsis*.

## Author Contributions

CH-V performed the experiments. JC and JM analyzed the data. JM and AA-M helped in writing the manuscript. JC conceived and designed the study and wrote the manuscript.

## Conflict of Interest Statement

The authors declare that the research was conducted in the absence of any commercial or financial relationships that could be construed as a potential conflict of interest.

## Footnotes

1 https://www.ncbi.nlm.nih.gov/geo/

## Supplementary Material

FIGURE S1 qPCR analysis of genes that are related to sulfate assimilation and that belong to module M1 of the sulfate co-expression network. For details, see the legend in **Figure 2**. Student’s *t*-test was performed to test the significant differences (*p* < 0.05) between FN and +/-S treatments. Significant changes are indicated with an asterisk.

FIGURE S2 Venn diagram showing the genes shared between nitrate- and sulfate-responsive genes. For this analysis, we considered only genes reported as differentially expressed in at least two different experiments in a previous study (Canales et al., 2014) or in the present study.

FIGURE S3 Selection of the Pearson’s correlation threshold for the construction of the sulfate co-expression network.

FIGURE S4 Overlap between genes and GO terms in our meta-analysis and the pioneering microarray experiments of Hirai et al. (2003), Nikiforova et al. (2003), and Maruyama-Nakashita et al. (2006).

TABLE S1 List of oligonucleotides used for qPCR analyses.

TABLE S2 List of sulfate-responsive genes ranked according to their consistency in the sulfate response.

TABLE S3 List of genes involved in the sulfate co-expression network ranked according to their number of connections.

TABLE S4 Gene Expression Omnibus accession numbers and descriptions of the samples included in **Figure 4B**.
