# Supplementary figures and images for: Integrative Transcriptomic Analysis Uncovers Novel Gene Modules That Underlie the Sulfate Response in Arabidopsis thaliana
Source: Front Plant Sci. 2018 Apr 10;9:470. doi: 10.3389/fpls.2018.00470 (PMC5902692; doi:10.3389/fpls.2018.00470)

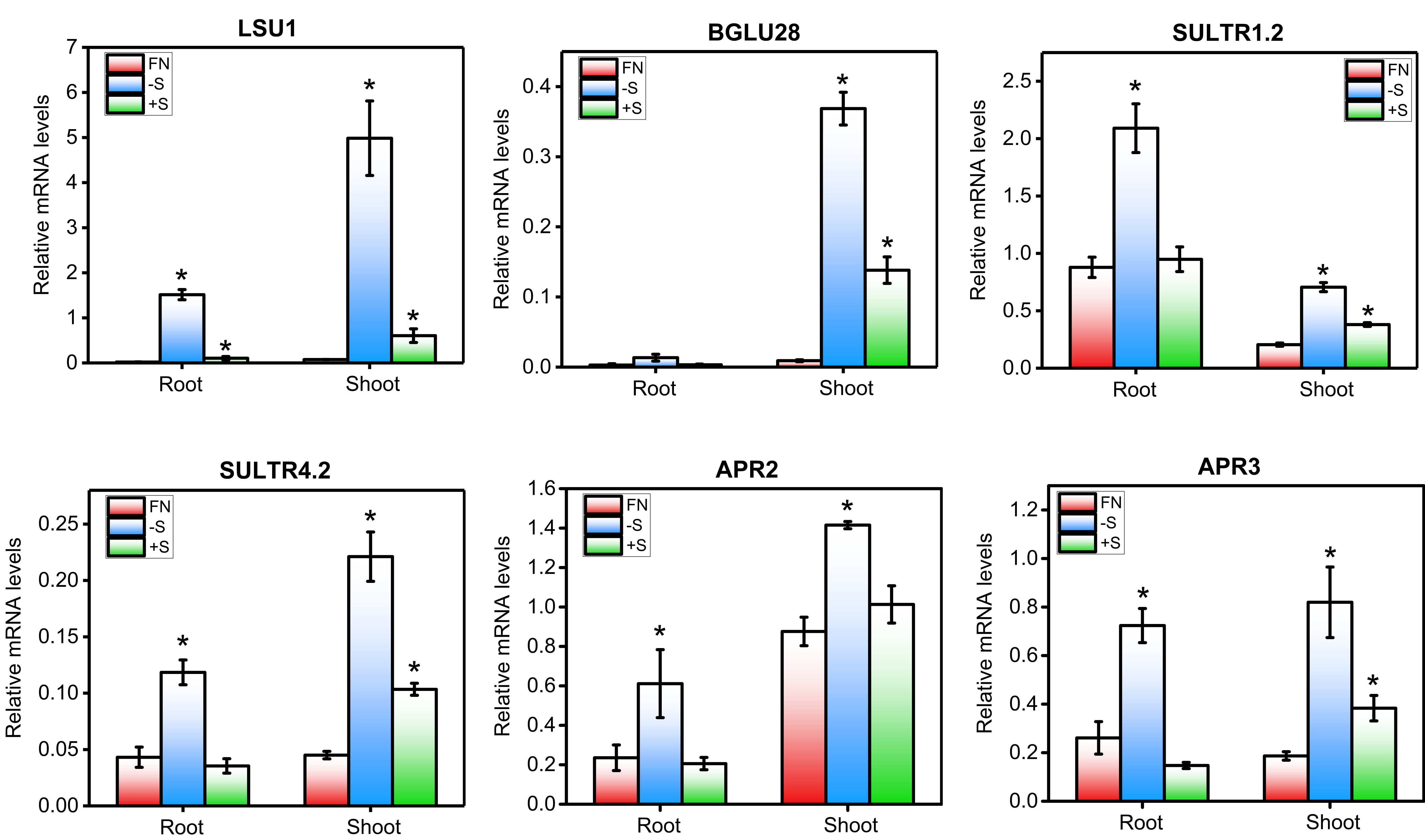

Supplement: FIGURE S1 — qPCR analysis of genes that are related to sulfate assimilation and that belong to module M1 of the sulfate co-expression network. For details, see the legend in Figure 2. Student’s t-test was performed to test the significant differences (p < 0.05) between FN and +/-S treatments. Significant changes are indicated with an asterisk. [file Image_1.JPEG]

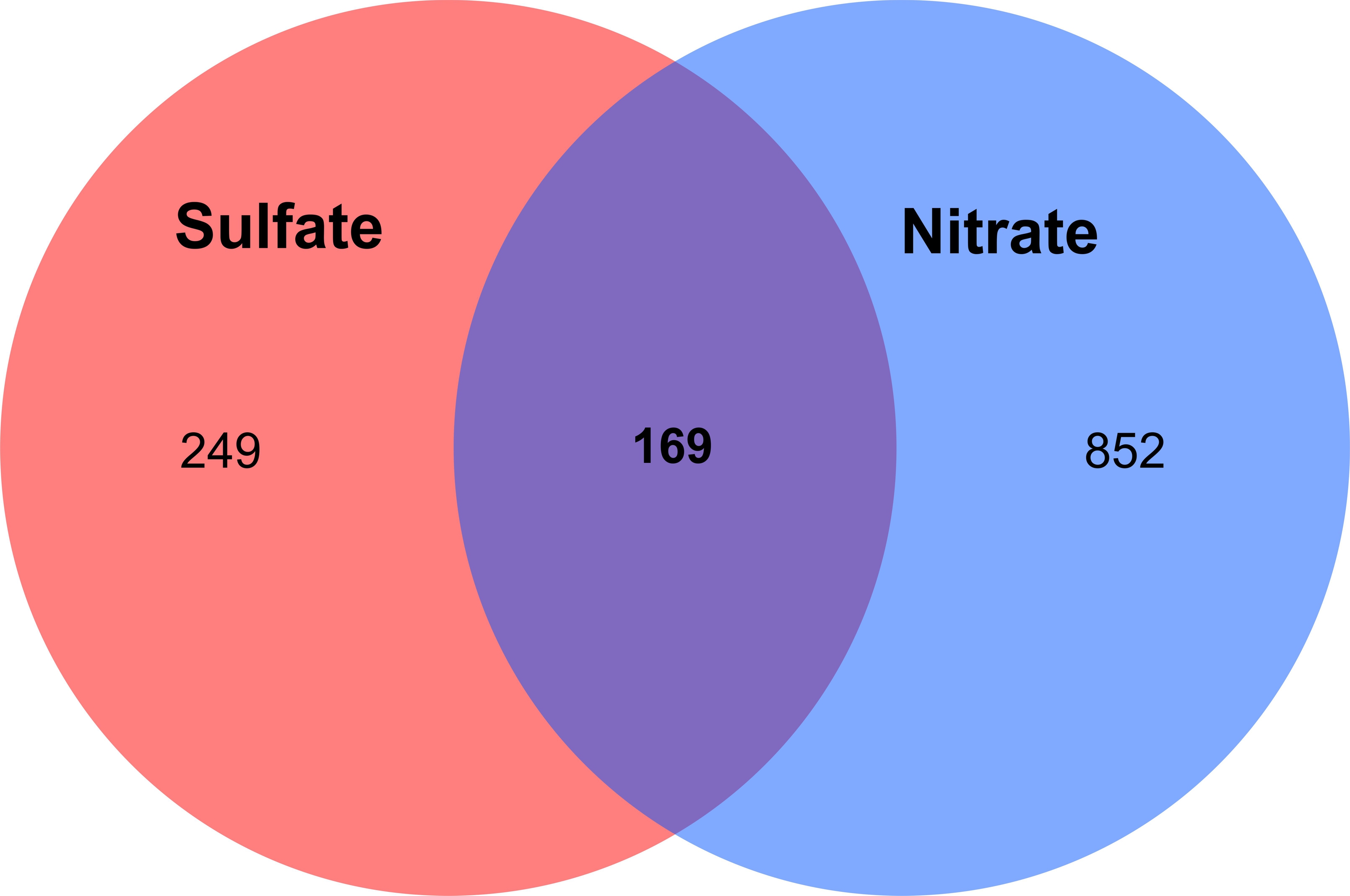

Supplement: FIGURE S2 — Venn diagram showing the genes shared between nitrate- and sulfate-responsive genes. For this analysis, we considered only genes reported as differentially expressed in at least two different experiments in a previous study (Canales et al., 2014) or in the present study. [file Image_2.JPEG]

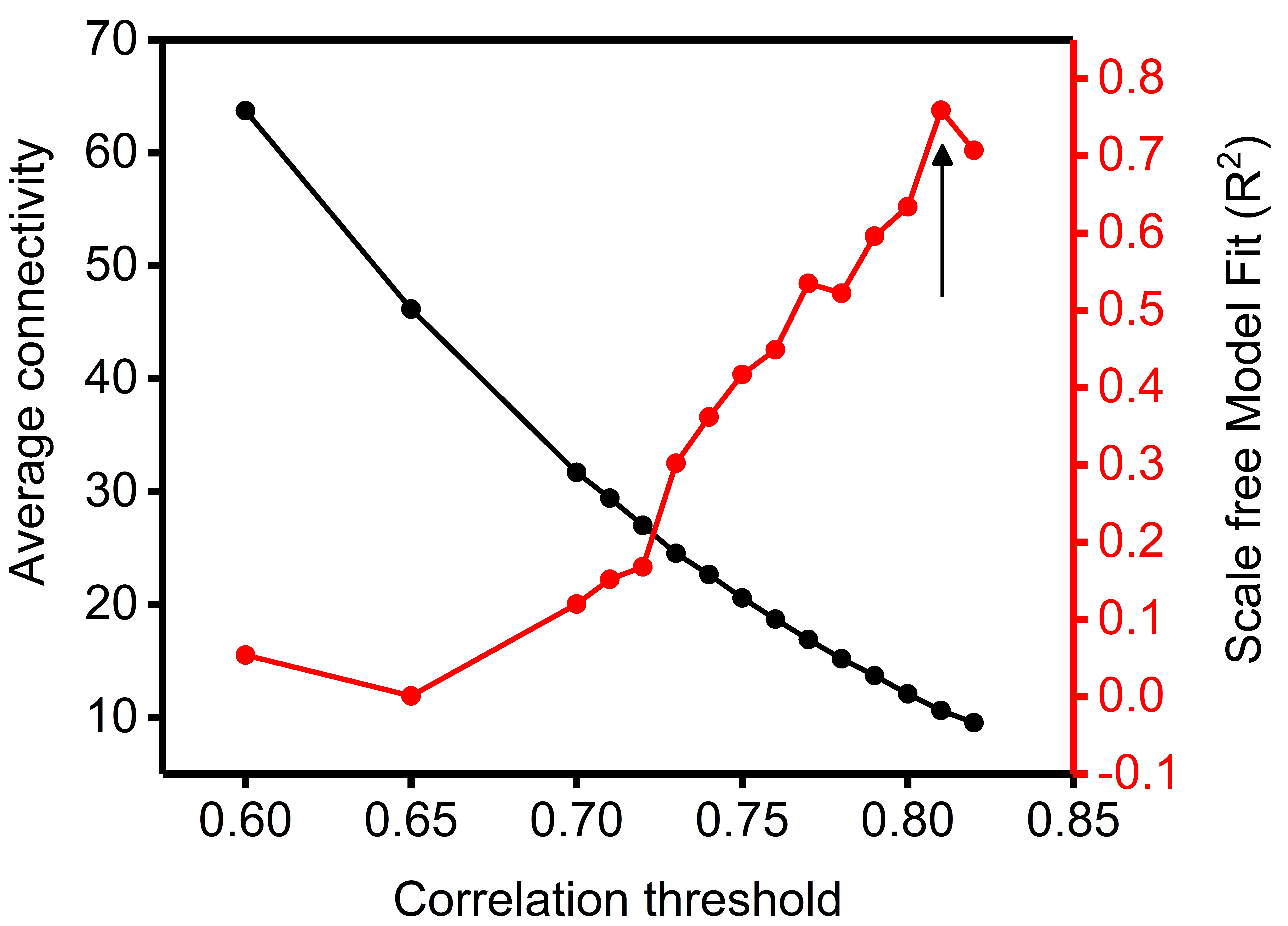

Supplement: FIGURE S3 — Selection of the Pearson’s correlation threshold for the construction of the sulfate co-expression network. [file Image_3.JPEG]

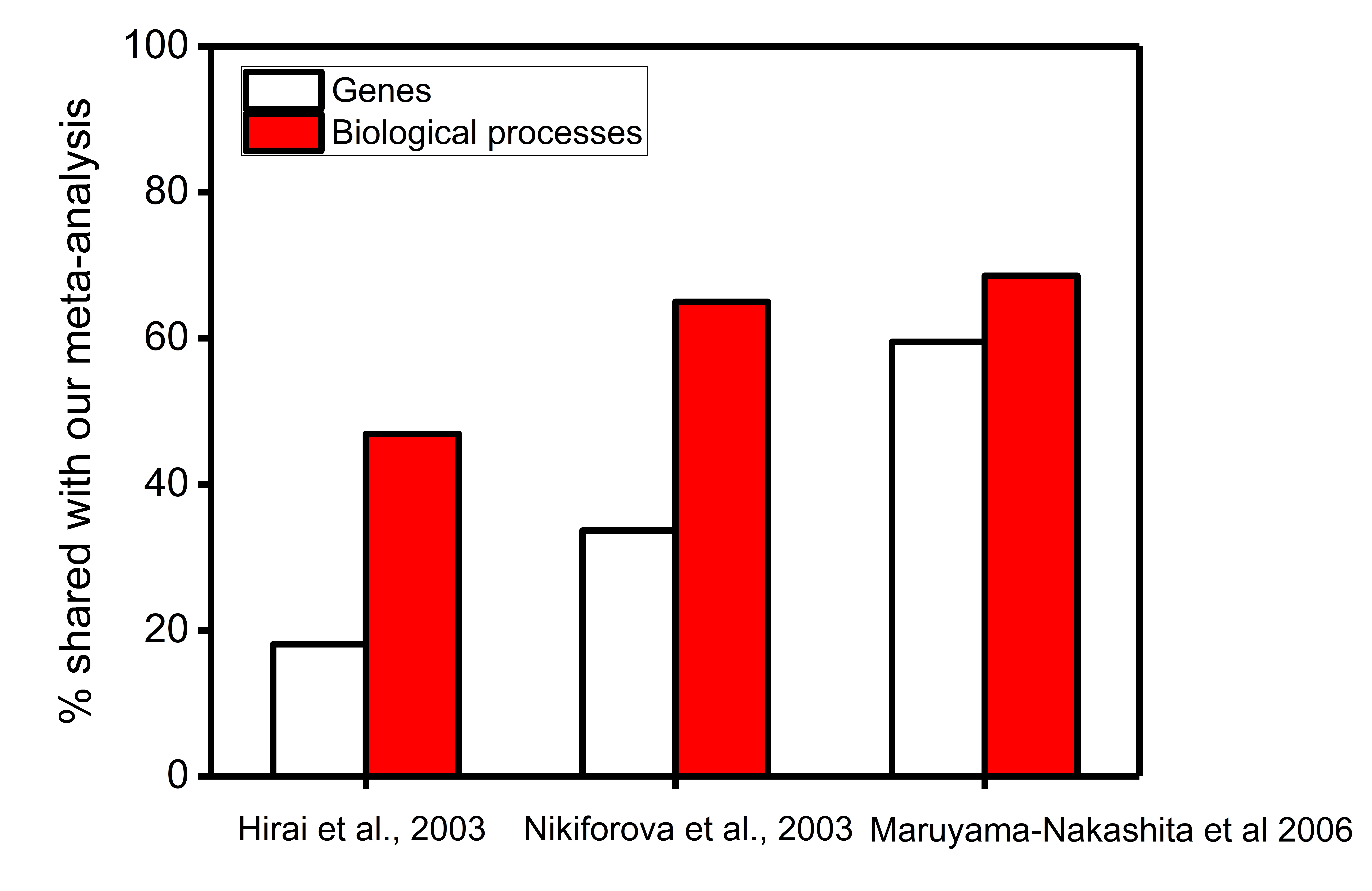

Supplement: FIGURE S4 — Overlap between genes and GO terms in our meta-analysis and the pioneering microarray experiments of Hirai et al. (2003), Nikiforova et al. (2003), and Maruyama-Nakashita et al. (2006). [file Image_4.JPEG]
